# Finite element analysis of the knee joint: a computational tool to analyze the combined behavior after treatment of torn ligaments and menisci in the human knee joint

**DOI:** 10.1051/sicotj/2024039

**Published:** 2024-10-31

**Authors:** Angelo V. Vasiliadis, Vasileios Giovanoulis, Alexandros Maris, Dimitrios Chytas, Konstantinos Katakalos, George Paraskevas, George Noussios, Aikaterini Vassiou

**Affiliations:** 1 Department of Orthopaedic Surgery, Sports Trauma Unit, St. Luke’s Hospital 55236 Panorama-Thessaloniki Greece; 2 Orthopaedic Surgery and Sports Medicine Department, FIFA Medical Center of Excellence, Croix-Rousse Hospital, Lyon University Hospital 69004 Lyon France; 3 Department of Anatomy, Faculty of Medicine, University of Thessaly 41334 Larissa Greece; 4 Department of Anatomy, School of Medicine, Faculty of Health Sciences, National and Kapodistrian University of Athens 11527 Athens Greece; 5 Department of Trauma and Orthopaedics, Royal Free Hospital NHS Trust W1W 5AQ London UK; 6 European University of Cyprus 2404 Engomi Nicosia Cyprus; 7 Basic Sciences Laboratory, Department of Physiotherapy, University of Peloponnese 23100 Sparta Greece; 8 Laboratory for Strength of Materials and Structures, Department of Civil Engineering, Aristotle University of Thessaloniki 54124 Thessaloniki Greece; 9 Department of Anatomy and Surgical Anatomy, School of Medicine, Faculty of Health Sciences, Aristotle University of Thessaloniki 54124 Thessaloniki Greece; 10 Department of Physical Education and Sports Sciences at Serres, Aristotle University of Thessaloniki 62122 Serres Greece

**Keywords:** Finite element analysis, Meniscal tear, Anterior cruciate ligament reconstruction, Posterior cruciate ligament reconstruction, Lateral tenodesis, Medial patellofemoral ligament reconstruction

## Abstract

Finite element analysis (FEA) is a fundamental tool that can be used in the orthopaedic world to simulate and analyze the behaviour of different surgical procedures. It is important to be aware that removing more than 20% of the meniscus could increase the shear stress in the cartilage and enlarge the risk of knee joint degeneration. In this fact, the maximal shear stress value in the medial cartilage increased up to 225% from 0.15 MPa to 0.5 MPa after medial meniscectomy. Also, meniscal root repair can improve meniscal biomechanics and potentially reduce the risk of osteoarthritis, even in cases of a loose repair. FEA has been used to better understand the biomechanical role of cruciate ligaments in the knee joint. ACLr with bone-patellar tendon-bone graft at 60 N of pretension and double-bundle PCLr were closer to that of a native knee in terms of biomechanics. The addition of a lateral extra-articular augmentation technique can reduce 50% of tibial translation and internal rotation, protecting the graft and minimizing the risk of re-rupture. Interestingly, anatomic and non-anatomic medial patellofemoral ligament reconstruction increased the pressure applied to the patellofemoral joint by increasing patellar contact pressure to 0.14 MPa at 30° of knee flexion using the semitendinosus as a graft. After all the advances in medical imaging technologies, future studies should take into consideration patient-specific data on both anatomy and mechanics, in order to better personalize the experimental model.

## Introduction

Finite element analysis (FEA) is a popular computerized method to virtual test and predict the reaction of different materials upon different ranges of forces [1]. FEA has become a fundamental tool in medical, veterinary and biological sciences in the last decades. In this aspect, FEA has also increased its popularity for the evaluation of biomechanics in orthopaedics [2, 3]. The FEA was first introduced in the field of orthopaedics by Brekelmans in 1972, by analyzing the mechanical behavior of human bones [4]. In 1983, Huiskes and Chao [5] recognized the potential of FEA as a valuable tool in basic research and orthopaedic biomechanics. In the early 1990 s, Beaupré et al. presented a time-dependent approach to simulate bone remodelling and predict changes in the proximal femur in the context of multiple loading conditions [6]. With the improvements in computational power and imaging capabilities, FEA is further applied to investigate more representative and complex bone models, at different scales from bone tissue to the cellular level [7].

The main advantage of the FEA is the real-time mode of approach, with the results being based on analysis of only one model [8, 9]. Also, FEA can predict how the use of different materials or surgical techniques (repair and reconstruction) can react when different forces are applied, while it helps to visualize the point of maximum stress [10]. Despite the various advantages of FEA, possible limitations are that FEA is a complex and informative simulation, as well as, more labour-intensive [2, 7]. The involvement of more specialized staff could overcome these problems. Also, FEA does not take into consideration changes in geometry after load application and changes in material properties [7]. However, this limitation could be addressed with the use of image-based models representative at different time points.

**Figure 1:**
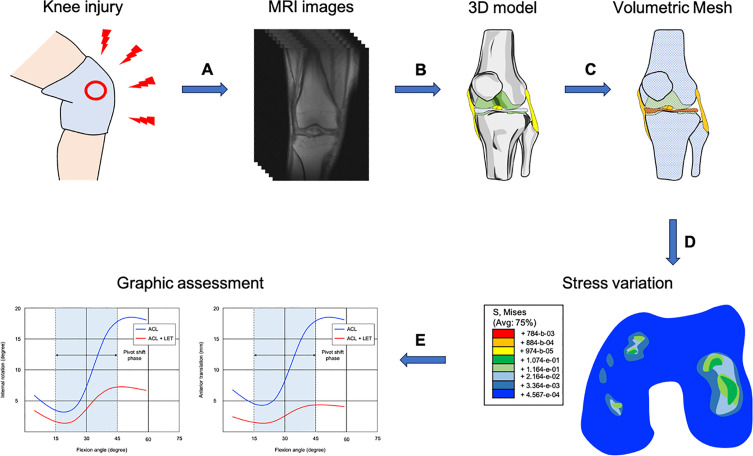
A graphical overview of the finite element analysis of the knee joint. A) A magnetic resonance imaging (MRI) is performed after knee injury. B) A three-dimensional (3D) model is created using computed tomography (CT) or/and MRI of the knee joint. C) The volumetric geometries of the knee joint are discretized for analysis. D) Contact stress behaviour of the femoral condyles after anterior cruciate ligament rupture. E) The profile of pivot shift phase after ACLr with and without LET.

The range of knee injuries that athletes and non-athletes may sustain varied from minimal injury, such as meniscal tear to more serious mechanical disruptions, like anterior cruciate ligament rupture and chronic degenerative injuries in the knee joint, where different kinds of operative techniques were proposed, to increase the longevity of the joint [11, 12]. In this aspect, FEA is a tool for better understanding the different treatment approaches for the knee (Figure 1). It can be also used as a tool, to investigate the effect of meniscectomies and meniscal repair on stress distribution in cartilage [13], to understand the mechanical behaviour of different techniques during anterior cruciate ligament reconstruction (ACLr) and posterior cruciate ligament reconstruction (PCLr) on the knee joint [14, 15], to evaluate the biomechanical performance of an additional procedure to the lateral aspect of the knee joint [16], as well as to assess the different surgical techniques for medial patellofemoral ligament reconstruction (MPFLr) on the patellofemoral contact pressure [17].

## FEA and meniscectomy

FEA shows that partial meniscectomy significantly alters the stress in the knee joint, leading to rapid surface damage wear and subsequent cartilage degeneration [13, 18]. More specifically, Vadher et al. [13] found that meniscectomy up to 20% produces little changes in shear stress in the cartilage, while the maximal shear stress in the cartilage increased up to 225%, from 0.15 MPa to 0.5 MPa, after 65% of the meniscus removal. They also noticed that the contact area between the femoral and tibial cartilage surface significantly increased, being more obvious with 20% of meniscus removal. The same findings were observed in cases of discoid meniscus and partial meniscectomy, where the contact stress increased dramatically with the width of the residual meniscus being less than 8 mm (40%) [18]. Moreover, Li et al. [19] demonstrated that the peak compression and the shear stress were also increased in the healthy tibiofemoral compartment, after enlargement of the meniscus tear region or/and meniscectomy in the opposite compartment. Interestingly, in cases of horizontal meniscal tear with further separation of the superior and inferior meniscal leaflet, the pattern of pressure and shear stress within the joint after resection of the superior leaflet seems to be closer to changes in pressure with those repaired with suture, making this option more reliable when meniscal repair is difficult (Table 1) [20].

**Table 1:** Summary of the major findings of the included studies regarding meniscal tears management.

Study	Material model	Experimental design	Conclusion
Vadher et al., 2006 [13]	Axisymmetric model	Shear stress in articular cartilage following various amounts of meniscus removed	Little changes in shear stress in the cartilage with up to 20% of meniscus removal, while meniscectomies > 20% increases drastically the shear stress in the cartilage
Liu et al., 2022 [18]	CT- and MRI-based knee model	Biomechanical behavior of the knee after partial meniscectomy in adults with complete discoid lateral meniscus	Meniscus width < 8 mm increases the contact stress significantly, while meniscus width of 8 to 10 mm provides a better biomechanical environment
Li et al., 2020 [19]	CT- and MRI-based knee model	Medial meniscus degeneration on the progress of the knee osteoarthritis	Degeneration of the medial meniscus leads to further increases of shear stress and also increases the shear stress in the healthy (lateral) compartment
Chen et al., 2023 [20]	MRI-based model	Internal force relationship of the knee after meniscus repair compared with different options of partial meniscus resection	Changes in shear stress within joint after resection of the superior leaflet of the meniscus is quite similar with meniscus repair and better when compared with the resection of the inferior leaflet of the meniscus
Wang et al., 2021 [21]	MRI-based model	Knee biomechanics after different repair methods for PRLM tears	Contact mechanics and kinematics of the knee after repair of PRLM can be similar to those of an intact knee joint
Xu et al., 2022 [22]	CT- and MRI-based knee model	Biomechanical characteristics of the knee joint after different patterns of PRMM tears versus meniscal repair	Loss of integrity of PRMM increase the load on the medial tibiofemoral articular cartilage, while meniscal repair restores the mechanical properties of the meniscus
Steineman et al., 2020 [23]	Imaged cadaveric knee specimens	Knee mechanics after non-anatomic placement of PRMM repair	Posterior placement of PRMM repairs results in greater changes to cartilage and meniscus mechanics, while anterior placement of PRMM repairs nearly restores contact tibiofemoral area
Steineman et al., 2022 [24]	Imaged cadaveric knee specimens	Knee mechanics after anatomic repair of PRMM compared with loosened repairs and untreated tears	Loosened repairs of PRMM can restore mechanics better than untreated meniscal root tears
Nabiyev et al., 2023 [25]	3D computer model	Knee biomechanics after oblique-vertical suture compared vertical suture technique for longitudinal meniscal tears	Oblique-vertical suture technique provide superior grip on the radial and circular fibers of the meniscus and a higher contact area compared to the classical vertical suture
Shriram et al., 2017 [26]	MRI-based cadaveric knee model	Cartilage contact pressures and distribution with the use of artificial meniscal implant versus meniscectomy	Anatomically shaped artificial implant induces lower peak cartilage contact pressure and reduces the cartilage regions loaded

Abbreviations: CT, computed tomography; MRI, magnetic resonance imaging; PRLM, posterior root lateral meniscus; PRMM, posterior root medial meniscus.

## FEA and meniscal repair

Meniscal repair is the best way to restore joint biomechanics and presumably offers a protective effect against the progression of OA [20]. Based on a recently published finite element analysis on meniscal repair, surgical repair of a torn meniscus can restore the loading profiles and joint kinematics to levels similar to those of an intact knee joint [21]. Especially with the posterior root repair of the meniscus, FEA studies have shown that the integrity of the posterior root plays a pivotal role, in achieving normal tibiofemoral contact mechanism [21, 22]. Anatomic fixation of the meniscal root to bone is important to restore normal biomechanics, while non-anatomic placement of meniscal root repairs may substantial negative effect on cartilage and meniscal function [23]. It is known from the literature that meniscal root repairs are susceptible to loosening, however, Steineman et al. [24] showed that loosened repairs would restore mechanics better than untreated meniscal root tears indicating the importance of anatomic repair (Table 1).

Various meniscal repair options have been reported in the literature, regarding the technique (all-inside, inside-out, outside-in), the suturing method (horizontal, vertical) and the number of sutures (single, double, multiple) [21, 27]. Wang et al. [21] showed that the double-stitch technique, for lateral meniscal posterior root avulsions, resulted in a significant decrease in joint contact pressure and contact stress, being more effective and leading to better clinical outcomes than the single-stitch technique. In addition, a 3D FEA model of a meniscus with a longitudinal tear revealed that the oblique-vertical suture repair technique provided a superior grip on the radial and circular fibres of the meniscus and higher contact area compared to vertical sutures [25]. It is important to note that while the posterior root meniscal repair restores the meniscal load transmission and the total contact area, a more posterior placement of the repair can induce meniscus extrusion and alter contact mechanics [23]. Interestingly, Shriram et al. [26] demonstrated that an anatomical-shaped artificial meniscal implant prevents higher contact pressures on articular cartilage and reduces the cartilage regions loaded when compared with meniscectomy (Table 1). In the same study, they demonstrated that the meniscal implant material stiffness plays a crucial role in contact pressures and implant displacement, with stiffness of 11 MPa restoring knee contact mechanics. Overall, current literature shows a positive effect of a meniscus repair on the functional outcome and progression of osteoarthritis [20, 23, 27].

## FEA and ACL/PCL reconstruction

FEA has also been used to investigate the factors that influence the success or failure of ACLr, including the graft tension, the tunnel placement, the type of fixation, the selection of graft and the additional lateral extra-articular procedure [14, 28]. Pena et al. [14] investigate the effect of graft stiffness and tensioning at different angles of knee joints and different tension loads. Their results showed that the anterior translation of the tibia after ACLr was closer to that of an intact knee when bone-patellar tendon-bone graft and 60 N of pretension were used. Regarding tunnel placement, Tampere et al. [29] found that the transtibial technique had a larger variance in tunnel placement on both the femoral and tibial side, with the anteromedial technique placing tunnels close to the anatomical centre of the ACL footprints. The graft fixation is essential for the longevity of ACLr, while the ideal fixation method should provide sufficient resistance to the daily forces, reduce post-operative stiffness and maintain the stability of the knee joint [28]. Abidin et al. [30] published a biomechanical analysis of the three different types of fixators for ACLr based on FEA and they found that cross-pin at the femur had the best stability. However, both the interference screw and the cortical button were found to be adequate for graft fixation (Table 2).

**Table 2:** Summary of the major findings of the included studies regarding ACL, PCL and ALL/LET.

Study	Material model	Experimental design	Conclusion
**ACL**			
Pena et al., 2005 [14]	MRI-based knee model	Graft stiffness and tensioning in ACLr at different knee flexion angles with three different grafts	Anterior translation of tibia was closer to that of an intact knee when BPTB graft and 60 N of pretension were used
Tampere et al., 2019 [29]	CT- and MRI-based knee model	Ability of the AM and TT techniques to achieve anatomical placement of femoral and tibial tunnel	AM technique provides shorter femoral tunnels and close to the anatomical footprint, while TT technique provides larger intra-articular, oval shaped hole and longer femoral tunnels
Abidin et al., 2021 [30]	CT-based knee model	Biomechanical effects of different types of fixators (cross-pin, interference screw, cortical button)	Cross-pin has optimum stability in terms of stress and strain at femoral site, while interference screw and cortical button provide adequate fixations for the graft
**PCL**			
Ramaniraka et al., 2005 [15]	CT- and MRI-based knee model	Effects of PCLr techniques (one bundle, two bundles) on the biomechanics of the knee joint	Resected PCL should be replaced to avoid compressive forces and cartilage degeneration, while both reconstruction techniques partially restore knee biomechanics
Yoon et al., 2010 [31]	CT-based knee model	Biomechanical evaluation of different PCLr techniques (one bundle, two bundle and two bundle augmentation)	Double bundle augmentation is superior regarding posterior and rotational stability and present lower stresses in the graft
Yang et al., 2023 [32]	CT-based knee model	Influence of TTA and PTS during PCLr	Anterior open-wedge HTO (+8° PTS), as well as a large TTA of 60° can affectively weaken the “killer turn” effect during PCLr
Wang et al., 2023 [33]	CT- and MRI-based knee model	Ideal femoral tunnel during PCLr to reduce peak stress of the graft	Femoral tunnel 5 mm distal and 5 mm anterior to the anatomical footprint can reduce the stress on the graft, without sacrifices the posterior stability of the knee
**ALL/LET**			
Risvas et al., 2024 [16]	MRI-based knee model	Interactions of ACLr combined LET on rotational stability of the knee	LET lead to a decrease in both external tibia rotation and posterior tibia translation, while larger values of tension may lead to over-constraint knee
Ugur et al., 2017 [34]	CT-based knee model	Reaction forces on tibia during internal rotation and ADT on both ACL and ALL	ALL is an important stabilizer against internal rotation of tibia, while ACL reflects an antagonist effect at 30° and higher flexion angles

Abbreviations: CT, computed tomography; MRI, magnetic resonance imaging; ACL, anterior cruciate ligament; ACLr, anterior cruciate ligament reconstruction; PCL, posterior cruciate ligament; PCLr, posterior cruciate ligament reconstruction; ALL/LET, anterolateral ligament/lateral extra-articular tenodesis; AM, anteromedial; TT, transtibial; BPTB, bone-patellar tendon-bone; TTA, tibial tunnel angle; PTS, posterior tibial slope; HTO, high tibia osteotomy; ADT, anterior drawer test.

Despite the relative importance of both ACL and PCL in knee joint function, PCL has received less attention than the more frequently injured ACL [15]. However, studies have evaluated the benefits of single- and double-bundle PCLr in terms of biomechanical outcomes [15, 31]. In a previous study, Yoon et al. [31] reported that the double-bundle augmentation PCL technique was more beneficial in restoring posterior and rotational stability. They also found that double-bundle PCL had lower ligament stress values, preventing secondary complications [31]. Furthermore, the importance of tunnel position at their anatomical locations is critical to providing superior post-operative stability on the knee joint [32, 33]. In particular, Yang et al. showed that anatomical placement and a large tibial tunnel angle of 60° can effectively weaken the “killer turn” effect during PCLr [32]. For the femoral side, Wang et al. [33] found that the placement of the femoral tunnel just 5 mm distal and 5 mm anterior to the footprint can reduce the stress of the graft and also reduce the “critical corner”, without eliminating the posterior stability of the knee joint (Table 2).

It should be noted that FEA has assisted regarding the reconstruction technique [29, 31], the graft tension [14], the type of fixation [30] and the proper tunnel placement [32, 33] in ligament reconstruction. However, FEA, an advanced computer-based method providing numerical solutions, requires further practical clinical verification, to confirm initial computational findings from ligament reconstruction. The main limitation of FEA is the reproduction of the viscoelastic properties of the ligament, limiting to accurate remodelling of the ligament behaviour. Ligament viscoelasticity decreases the load graft tension and stiffness, while it plays an important role in the final graft fixation [14]. The simplification of ligament modelling by using linear, isotropic and homogenous elements may affect the predictions from real-life conditions [30, 32]. Nevertheless, ligament simplification, by using linear spring elements, has been used in the literature with acceptable accuracy outcomes [30, 32].

## FEA and lateral extra-articular augmentation techniques (LEAT)

The anterolateral ligament (ALL) is an important structure of the lateral knee, playing a crucial role in dynamic daily activities [35]. An FEA study based on a three-dimensional solid knee model found that the ALL is a secondary stabilizer of the knee joint, especially against the internal rotation of the knee at a higher of 30° of flexion [34]. It has been proven that both ligaments, ALL and ACL, act as secondary stabilizers to each other under dynamic conditions, especially during internal tibial rotation [35]. In this fact, different extra-articular augmentation techniques have been used to protect and minimize the risk of re-rupture after ACLr. Among them, ALL reconstruction and lateral extra-articular tenodesis (LET) with the use of an iliotibial band are the most common lateral augmentation procedures [36].

Studies have shown that both techniques increase rotational stability by minimizing the anterior translation and internal rotation while eliminating the risk of graft failure [16, 37]. Specifically, tibial anterior translation was less than 4 mm for the intact knees and knees with ALL reconstruction, while the translation was up to 10 mm in deficient knees [37]. In the same direction, intact and reconstructed knees revealed an internal rotation between 6° and 12° depending on flexion angle, with internal rotation being up to 20° in deficient knees [37]. In addition, regarding the combined ACLr and LET, the posterior translation during the pivot shift test showed up to 3.5 mm of translation, while ACLr alone yielded up to 5.5 mm of translation [16]. Therefore, it can be easily concluded that LEAT reduces approximately 50% of tibial translation and internal rotation. On the other hand, this behaviour of LEAT can lead to over-constraint of the lateral compartment of the knee joint. Risvas et al. [16] showed that LET leads to over-constrained knee behaviour regarding external tibial rotation (Table 2), while this effect on the range of motion of the knee and advancement of osteoarthritis is still controversial. Furthermore, in order to address the problem of instability without risking over-constraint the knee, Thaunat et al. [37] proposed a more postero-proximal femoral attachment for ALL reconstruction. This procedure minimizes the tibiofemoral contact forces and decreases the risk of over-constraint the lateral tibiofemoral compartment.

Currently, the type of treatment is based on the long-term objectives and whether the patient wishes to continue demanding activities and sports [16]. Both ALL reconstruction and LET have been shown safe and effective, minimizing the risk of graft failure. Thus, the addition of a LEAT seems to be a critical factor that affects the long-term outcomes [16, 36, 37].

## FEA and MPFL reconstruction

Traumatic patellar dislocation is a common sports-related knee injury that occurs most often in young, active patients under the age of 20 years [38]. Considering that almost 100% of patients with patellar dislocation suffer from MPFL rupture [39], MPFL reconstruction may be a reliable surgical treatment for a first-time episode [40, 41]. Studies have shown an increase in the patellofemoral contact pressures after both anatomic and non-anatomic MPFLr [17, 42]. According to Sanchis-Alfonso et al. [17], the average patella contact pressure increased to 6.55 MPa and 14.74 MPa at 30° of knee flexion for anatomic and non-anatomic MPFLr, respectively. In the same study, authors observed an increase in the patellofemoral contact pressure to 2.17 MPa at 0° and 0.14 MPa at 30° with the use of semitendinosus as a graft compared to normal native knee (0.18 MPa at 0° and 0.016 MPa at 30°) [17]. However, Kheir et al. [43] have shown that concomitant lateral retinaculum release with MPFLr in knees with tibial tuberosity-trochlea groove of 12 mm may result in approximately 40% decrease in contact pressure and contact area. This may have a negative impact, increasing the lateral patellar displacement with increased knee flexion, which may predispose to the risk of lateral patellar instability. Moreover, Sanchis-Alfonso et al. [44] found that the dynamic MPFLr resulted in lower patellar contact pressure from 0° to 30° of knee flexion compared to static reconstruction. They found that the pressure in dynamic reconstructions was similar compared with an intact knee, minimizing the risk of patellofemoral osteoarthritis in the long term (Table 3) [44].

**Table 3:** Summary of the major findings of the included studies regarding MPFL.

Study	Material model	Experimental design	Conclusion
Watson et al., 2015 [42]	MRI-based knee model	Lateral force displacement of the patella after MPFLr	MPFLr increases the patella lateral restraining force, while a more anterior placement to the femoral anatomical insertion could increase the contact force and area
Kheir et al., 2022 [43]	MRI-based knee model	Knee biomechanics after lateral retinaculum release combined with MPFLr	Release of the lateral retinaculum combined MPFLr decrease patellofemoral contact pressure/area and increase lateral patella displacement starting at 20° knee flexion
Sanchis-Alfonso et al., 2019 [44]	CT-based knee model	PF biomechanics after different MPFL fixation techniques	Patellar contact pressures after dynamic MPFLr were similar of the native knee, whereas static MPFLr resulted in greater pressures, potentially increasing the risk of PF OA in long term
Watson et al., 2017 [45]	MRI-based knee model	Biomechanical effects of patella alta on contact pressures within PF joint after MPFLr	Patella alta after MPFLr decreases lateral restraining force and PF contact area, while increases PF contact pressures
Wei et al., 2024 [46]	CT-based knee model	Explore the more suitable MPFLr strategies for young patients with open physis	A distally-located femoral graft insertion site during MPFLr demonstrates positive outcomes

Abbreviations: CT, computed tomography; MRI, magnetic resonance imaging; MPFL, medial patellofemoral ligament; MPFLr, medial patellofemoral ligament reconstruction; PF, patellofemoral; OA, osteoarthritis.

Considering the femoral insertion, Watson et al. [45] found increased maximum patellofemoral contact pressures following MPFLr when the femoral insertion site was placed anterior and distal to the anatomic insertion. The choice of the femoral insertion in MPFLr for young patients with open physis is also crucial [46]. A femoral graft insertion site, which is located distally from the growth plate, demonstrates positive outcomes, while an FEA may increase post-operative stability and minimize risks associated with operative intervention (Table 3) [46].

Despite the significant findings from the literature over the last decade, the potential limitations of the MPFLr and FEA are that the patellofemoral joint anatomy is a unique structure and presents a high variability. Most of the studies include models, which are based on a normal knee without taking into consideration factors for patellar instability, such as trochlea dysplasia, lateralization of the tibial tubercle and patella height [43]. In addition, there is a problem among studies, which are trying to distinguish the soft tissues, the cartilage and the bone, where the patellofemoral joint was reconstructed based on CT scans [17, 44, 46]. In these studies, cartilage thickness was estimated by taking a fixed measure into account [44, 46].

## Conclusions

Orthopaedic surgery is a topic where FEA can assist in different parts. FEA is a computer simulation technique and provides many benefits against real models. It is used to predict the behaviour of an anatomical structure, a surgical technique and an individualized treatment option under different external forces, such as stress and strain. This method is reproducible without ethical limitations and may be very helpful in reproducing a surgical procedure and allow the surgeons to simulate how this procedure could respond before being applied to patients. Although FEA has been widely applied in the orthopaedic world for more than four decades, many things should also be planned and investigated, to improve the accuracy and the effectiveness of this method. In the future, FEA models may combine MRI-CT data allowing more precise extraction of cartilage thickness and also determining patient-specific characteristics, which are related to the integrity of the model.

## Data Availability

Data are available on request from the authors.

## References

[1] Welch-Phillips A, Gibbons D, Ahern DP, Butler JS (2020) What is finite element analysis? Clin Spine Surg 33, 323–324. 32675684 10.1097/BSD.0000000000001050

[2] Pfeiffer FM (2016) The use of finite element analysis to enhance research and clinical practice in orthopaedics. J Knee Surg 29, 149–158. 26745731 10.1055/s-0035-1570114

[3] Batailler C, Shatrov J, Schmidt A, Servien E, Puch JM, Lustig S (2021) Similar stress repartition for a standard uncemented collared femoral stem versus a shortened collared femoral stem. SICOT-J 7, 58. 34797213 10.1051/sicotj/2021061 PMC8603923

[4] Brekelmans W, Poort H, Slooff T (1972) A new method to analyse the mechanical behaviour of skeletal parts. Acta Orthop Scand 43, 301–317. 4651051 10.3109/17453677208998949

[5] Huiskes R, Chao E (1983) A survey of finite element analysis in orthopedic biomechanics: the first decade. J Biomech 16, 385–409. 6352706 10.1016/0021-9290(83)90072-6

[6] Beaupré GS, Orr TE, Carter DR (1990) An approach for time-dependent bone modeling and remodeling-theoretical development. J Orthop Res 8, 651–661. 2388105 10.1002/jor.1100080506

[7] Meslier QA, Shefelbine SJ (2023) Using finite element modeling in bone mechanoadaptation. Curr Osteoporos Rep 21, 105–116. 36808071 10.1007/s11914-023-00776-9 PMC10105683

[8] Chang S, Liu K, Yang M, Yuan L (2022) Theory and implementation of sub-model method in finite element analysis. Heliyon 8, e11427. 36387453 10.1016/j.heliyon.2022.e11427 PMC9647488

[9] Xie H, Song J, Gao B, Zhong Y, Gu C, Choi K-S (2021) Finite-element Kalman filter with state constraint for dynamic soft tissue modeling. Comput Biol Med, 135 104594. 34182332 10.1016/j.compbiomed.2021.104594

[10] Srirekha A, Bashetty K (2010) Infinite to finite: an overview of finite element analysis. Indian J Dent Res 21, 425–432. 20930357 10.4103/0970-9290.70813

[11] Seil R, Becker R (2016) Time for a paradigm change in meniscal repair: save the meniscus! Knee Surg Sports Traumatol Arthrosc 24, 1421–1423. 27107860 10.1007/s00167-016-4127-9

[12] Giovanoulis V, Schmidt A, Vasiliadis AV, Koutserimpas C, Batailler C, Lustig S, Servien E (2024) Prior medial meniscus arthroscopy is not associated with worst functional outcomes in patients undergoing primary total knee arthroplasty: A retrospective single-center study with a minimum follow-up of 5 years. SICOT-J 10, 5. 38240731 10.1051/sicotj/2024001 PMC10798227

[13] Vadher SP, Nayeb-Hashemi H, Canavan PK, Warner GM (2006) Finite element modeling following partial meniscectomy: effect of various size of resection. Conf Proc IEEE Eng Med Biol Soc 2006, 2098–2101. 17946937 10.1109/IEMBS.2006.259378

[14] Pena E, Martinez MA, Calvo B, Palanca D, Doblare M (2005) A finite element simulation of the effect of graft stiffness and graft tensioning in ACL reconstruction. Clin Biomech (Bristol, Avon) 20, 636–644. 15927737 10.1016/j.clinbiomech.2004.07.014

[15] Ramaniraka NA, Terrier A, Theumann N, Siegrist O (2005) Effects of the posterior cruciate ligament reconstruction on the biomechanics of the knee joint: a finite element analysis. Clin Biomech (Bristol, Avon) 20, 434–442. 15737452 10.1016/j.clinbiomech.2004.11.014

[16] Risvas K, Stanev D, Moustakas K (2024) Can lateral tenodesis improve the rotational stability of the ACL reconstruction? A finite element analysis PLoS One 19, e0293161. 38412190 10.1371/journal.pone.0293161 PMC10898738

[17] Sanchis-Alfonso V, Alastruey-Lopez D, Ginovart G, Montesinos-Berry E, Garcia-Castro F, Ramirez-Fuentes C, Carles Monllau J, Alberich-Bayarri A, Angeles Perez M (2019) Parametric finite element model of medial patellofemoral ligament reconstruction model development and clinical validation. J Exp Orthop 6, 32. 31278510 10.1186/s40634-019-0200-x PMC6611858

[18] Liu W, Sun X, Liu W, Liu H, Zhai H, Zhang D, Tian F (2022) Finite element study of a partial meniscectomy of a complete discoid lateral meniscus in adults. Med Eng Phys, 107, 103855. 35914995 10.1016/j.medengphy.2022.103855

[19] Li L, Yang L, Zhang K, Zhu L, Wang X, Jiang Q (2020) Three-dimensional finite-element analysis of aggravating medial meniscus tears on knee osteoarthritis. J Orthop Translat 20, 47–55. 31908933 10.1016/j.jot.2019.06.007 PMC6939112

[20] Chen H, Liu L, Zhang Y (2023) Finite element analysis of the knee joint stress after partial meniscectomy for meniscus horizontal cleavage tears. BMC Musculoskelet Disord 24, 744. 37726679 10.1186/s12891-023-06868-y PMC10508030

[21] Wang JY, Qi YS, Bao HRC, Xu YS, Wei BG, Wang YX, Ma BX, Zhou HW, Lv F (2021) The effects of different repair methods for posterior root tear of the lateral meniscus on the biomechanics of the knee: a finite element analysis. J Orthop Surg Res 16, 296. 33952275 10.1186/s13018-021-02435-0 PMC8097866

[22] Xu Z, Li Y, Rao J, Jin Y, Huang Y, Xu X, Liu Y, Tian S (2022) Biomechanical assessment of disease outcome in surgical interventions for medial meniscal posterior root tears: a finite element analysis. BMC Musculoskelet Disord 23, 1093. 36517757 10.1186/s12891-022-06069-z PMC9749342

[23] Steineman BD, LaPrade RF, Haut Donahue TL (2020) Nonanatomic placement of posteromedial meniscal root repairs: A finite element study. J Biomech Eng 142, 081004. 31901167 10.1115/1.4045893

[24] Steineman BD, LaPrade RF, Haut Donahue TL (2022) Loosening of posteromedial meniscal root repairs affects knee mechanics: A finite element study. J Biomech Eng, 144, 051003. 34817052 10.1115/1.4053100

[25] Nabiyev E, Baizakov A, Kashikova K, Askerov R, Argynbayev Z, Bissaliyev B (2023) A new approach to arthroscopic stitching of the knee joint meniscus: A mathematical justification. Med J Islam Repub Iran 37, 108. 38145180 10.47176/mjiri.37.108 PMC10744200

[26] Shriram D, Kumar GP, Cui F, Lee YHD, Subburaj K (2017) Evaluating the effects of material properties of artificial meniscal implant in the human knee joint using finite element analysis. Sci Rep 7, 6011. 28729605 10.1038/s41598-017-06271-3 PMC5519683

[27] Beaufils P, Pujol N (2018) Meniscal repair: Technique. Orthop Traumatol Surg Res 104, S137–S145. 29175557 10.1016/j.otsr.2017.04.016

[28] Benos L, Stanev D, Spyrou L, Moustakas K, Tsaopoulos DE (2020) A review on finite element modeling and simulation of the anterior cruciate ligament reconstruction. Front Bioeng Biotechnol 8, 967. 32974307 10.3389/fbioe.2020.00967 PMC7468435

[29] Tampere T, Devriendt W, Cromheecke M, Luyckx T, Verstraete M, Victor J (2019) Tunnel placement in ACL reconstruction surgery: smaller inter-tunnel angles and higher peak forces at the femoral tunnel using anteromedial portal drilling – a 3D and finite element analysis. Knee Surg Sports Traumatol Arthrosc 27, 2568–2576. 30406406 10.1007/s00167-018-5272-0

[30] Abidin NAZ, Wahab AHA, Rahim RAA, Kadir MRA, Ramlee MH (2021) Biomechanical analysis of three different types of fixators for anterior cruciate ligament reconstruction via finite element method: a patient-specific study. Med Biol Eng Comput 59, 1945–1960. 34392448 10.1007/s11517-021-02419-6

[31] Yoon KH, Kim Yh, Ha JH, Kim K, Park WM (2010) Biomechanical evaluation of double bundle augmentation of posterior cruciate ligament using finite element analysis. Clin Biomech (Bristol, Avon) 25, 1042–1046. 20817365 10.1016/j.clinbiomech.2010.07.014

[32] Yang F, Yokoe T, Ouchi K, Tajima T, Chosa E (2023) Influence of the tibial tunnel angle and posterior tibial slope on “killer turn” during posterior cruciate ligament reconstruction: A three-dimensional finite element analysis. J Clin Med 12, 805. 36769453 10.3390/jcm12030805 PMC9917875

[33] Wang B, Ye Y, Yao L, Wei A, Huang X, Wang Z, Yu X (2023) Different femoral tunnel placement in posterior cruciate ligament reconstruction: a finite element analysis. BMC Musculoskelet Disord 24, 93. 36737713 10.1186/s12891-023-06161-y PMC9898916

[34] Ugur L (2017) Comparison of reaction forces on the anterior cruciate and anterolateral ligaments during internal rotation and anterior drawer forces at different flexion angles of the knee joint. Int J Med Robot 13, e1815. 10.1002/rcs.1815 28251769

[35] Kang KT, Koh YG, Park KM, Choi CH, Jung M, Cho H, Kim SH (2022) Effects of the anterolateral ligament and anterior cruciate ligament on knee joint mechanics: A biomechanical study using computational modeling. Orthop J Sports Med 10, 23259671221084970. 35400144 10.1177/23259671221084970 PMC8988680

[36] Beckers L, Vivacqua T, Firth AD, Getgood AMJ (2021) Clinical outcomes of contemporary lateral augmentation techniques in primary ACL reconstruction: a systematic review and meta-analysis. J Exp Orthop, 8, 59. 34383156 10.1186/s40634-021-00368-5 PMC8360253

[37] Thaunat M, Ingale PS, De Guise J, Dumas R, Blache Y (2020) The effect of anterolateral ligament reconstruction on knee constraint: A computer model-based simulation study. Knee 27, 1228–1237. 32711886 10.1016/j.knee.2020.05.006

[38] Tsai CH, Hsu CJ, Hung CH, Hsu HC (2012) Primary traumatic patellar dislocation. J Orthop Surg Res 7, 21. 22672660 10.1186/1749-799X-7-21 PMC3511801

[39] Krebs C, Tranovich M, Andrews K, Ebraheim N (2018) The medial patellofemoral ligament: Review of the literature. J Orthop 15, 596–599. 29881201 10.1016/j.jor.2018.05.004 PMC5990246

[40] Vezole L, Gunst S, Gras LL, Shatrov J, Mertbakan O, Lustig S, Servien E (2024) What is the best fixation method in medial patellofemoral ligament reconstruction? A biomechanical comparison of common methods for femoral graft attachment. SICOT-J, 10, 7. 38334592 10.1051/sicotj/2024004 PMC10854485

[41] Yoo JD, Huh MH, Lee CW, Roh YH, D’Lima DD, Shin YS (2023) Medial patellofemoral ligament reconstruction appears to be a better treatment than repair, proximal realignment, or conservative management for primary patellar dislocation: A network meta-analysis. Medicine (Baltimore), 102, e35251. 37773862 10.1097/MD.0000000000035251 PMC10545352

[42] Watson NADV, Duchman KR, Bollier MJ, Grosland NM (2015) A finite element analysis of medial patellofemoral ligament reconstruction. Iowa Orthop J 35, 13–19. 26361439 PMC4492131

[43] Kheir N, Salvatore G, Berton A, Orsi A, Egan J, Mohamadi A, DeAngelis JP, Ramappa AJ, Longo UG, Denaro V, Nazarian A (2022) Lateral release associated with MPFL reconstruction in patients with acute patellar dislocation. BMC Musculoskelet Disord 23, 139. 35148741 10.1186/s12891-022-05013-5 PMC8832651

[44] Sanchis-Alfonso V, Ginovart G, Alastruey-Lopez D, Montesinos-Berry E, Monllau JC, Alberich-Bayarri A, Perez MA (2019) Evaluation of patellar contact pressure changes after static versus dynamic medial patellofemoral ligament reconstructions using finite element model. J Clin Med 8, 2093. 31805708 10.3390/jcm8122093 PMC6947356

[45] Watson NA, Duchman KR, Grosland NM, Bollier MJ (2017) Finite element analysis of patella alta: A patellofemoral instability model. Iowa Orthop J 37, 101–108. 28852343 PMC5508303

[46] Wei WQ, Sha L, Zhan S, Zhang RZ (2024) Finite element analysis of MPFL reconstruction in a pediatric patient: A case report. Asian J Surg 12, S1015-9584(24)01197-7. 10.1016/j.asjsur.2024.05.287 38871610

